# Stability and complexity in model meta-ecosystems

**DOI:** 10.1038/ncomms12457

**Published:** 2016-08-24

**Authors:** Dominique Gravel, François Massol, Mathew A. Leibold

**Affiliations:** 1 Département de biologie, Université de Sherbrooke, 2500 Boul. De l'Université, Sherbrooke, Quebec, Canada J1K 2R1; 2 CNRS, Université de Lille - Sciences et Technologies, UMR 8198 Evo-Eco-Paleo (EEP), SPICI group, F-59655 Villeneuve d'Ascq, France; 3 UMR 5175 CEFE—Centre d'Ecologie Fonctionnelle et Evolutive (CNRS), 1919 Route de Mende, F-34293 Montpellier, France; 4 Department of Integrative Biology, University of Texas at Austin, Austin, Texas 78712, USA

## Abstract

The diversity of life and its organization in networks of interacting species has been a long-standing theoretical puzzle for ecologists. Ever since May's provocative paper challenging whether ‘large complex systems [are] stable' various hypotheses have been proposed to explain when stability should be the rule, not the exception. Spatial dynamics may be stabilizing and thus explain high community diversity, yet existing theory on spatial stabilization is limited, preventing comparisons of the role of dispersal relative to species interactions. Here we incorporate dispersal of organisms and material into stability–complexity theory. We find that stability criteria from classic theory are relaxed in direct proportion to the number of ecologically distinct patches in the meta-ecosystem. Further, we find the stabilizing effect of dispersal is maximal at intermediate intensity. Our results highlight how biodiversity can be vulnerable to factors, such as landscape fragmentation and habitat loss, that isolate local communities.

## Abstract (web summary)

The mechanisms allowing highly biodiverse ecosystems to remain stable are poorly understood. Here, Gravel and colleagues provide theoretical evidence that dispersal of organisms and material modifies species interactions and thus enables highly biodiverse communities to exist.

## Graphical abstract

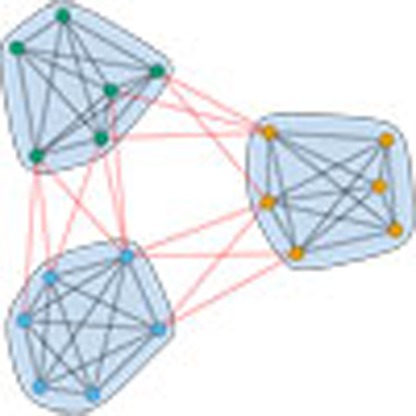

The diversity of species within natural communities and the complexity of their interactions in nature have fascinated ecologists. In particular, the question of whether large complex ecosystems should be stable has been a pervading theme in ecology, both theoretically and empirically. More than 40 years ago, and contrary to previous intuition^1^,^2^, May predicted that diversity and complexity should destabilize ecosystems^3^,^4^. The common observation of highly diverse communities has consequently been a major puzzle for ecologists. Ensuing work has focused in part on alternate definitions of ecosystem stability^5^,^6^,^7^,^8^,^9^, and on more complex hypotheses based on different mechanisms, such as allometric correlations, adaptive foraging or stage structure, to explain this so-called ‘complexity–stability' paradox^10^,^11^,^12^,^13^,^14^,^15^,^16^. Explaining very diverse communities nonetheless remains elusive and keeps attracting the attention of ecologists.

May^3^,^4^ studied the dynamical properties of randomly assembled ecosystems. He modelled them using the Jacobian matrix, which describes the pairwise effects of one species on another and could be used to investigate the rate at which the ecosystem returns to the equilibrium following a disturbance (as measured by the leading eigenvalue of the Jacobian). He found that stability should decrease with the number of species and interactions between them. By varying the number of species *S*, the connectance *c* (the proportion of potential interactions among all pairs of species that are realized), the s.d. of interspecific interaction strength (*σ*) and the average intraspecific interaction strength (*m*), this theory indicates that for a community to be stable, it must respect the following inequality^3^,^16^:


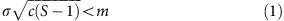


In other words, there is a three-dimensional trade-off between species diversity, connectance and the interaction strength that allows systems to be stable. Inequality (1) stems from the fact that for random Jacobian matrices following the circular law^17^, the empirical spectral distribution of eigenvalues (that is, their location in the complex plane) forms a disk centred at the average feedback coefficient among species (that is, around *−m*) with a radius proportional to the s.d. of the Jacobian matrix (here, 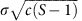), and thus comprising eigenvalues with positive real part (and thus unstable local equilibria) only when inequality (1) is not verified. In practice, this means that ecosystems are likely to be relatively simple, so that the common observation of hundreds to thousands of species in local communities is difficult to explain.

Since then, numerous hypotheses to explain this paradox have been invoked^8^,^9^,^18^,^19^. Most, if not all of them, have some empirical support, but their absolute or relative importance is not well understood. One of the most important possibilities is that meta-ecosystems, defined as local ecosystems connected by spatial exchanges of individuals, energy and material^20^,^21^,^22^, could be more stable than their isolated components^23^. Spatial flows among local ecosystems are ubiquitous in nature^24^, connecting not only similar habitats (for example, patches of forest and lakes) but also very different ones (for example, ocean/island, stream/forest and benthic/pelagic). However, the strength and sign of this effect on stability is not clear. Most studies so far have focused on small food web modules^25^ or have been conducted with different measurements of stability that are not directly comparable to May's local stability^25^,^26^.

To understand how spatial flows among local ecosystems might stabilize dynamics at the meta-ecosystem scale, we expand the approach taken by May^3^,^4^ to look at the structure of the Jacobian matrix of the meta-ecosystem when placed in a spatial context^27^. We considered the technique of local stability analysis, along with random interaction matrices, as a starting point to facilitate the comparison with May's criterion. Although this approach has been criticized for some of its assumptions^5^,^19^, our goal is to allow a direct comparison of stability on May's result with and without spatial effects.

Jacobian matrices are obtained by linearizing the system of equations describing the dynamics of all species making an ecosystem at equilibrium. A Jacobian matrix thus describes the direct interactions among all pairs of populations near this equilibrium. Stability is assessed from its largest eigenvalue, and the system is stable when the real part of the largest eigenvalue is negative. Extending the approach of May, the Jacobian matrix **J** of a meta-ecosystem can be expressed as the sum of three matrices (Fig.1):


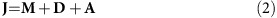


where **M** is a deterministic diagonal matrix representing intraspecific density dependence, with value *−m*_*i*_ along the diagonal and 0 in the rest of the matrix; **D** is a deterministic matrix representing dispersal among patches; and **A** is the collection of local Jacobian matrices, arranged as diagonal blocks describing interspecific interactions within each local community. Following May's approach, we assume that the sub-matrices **A** are stochastic with entries subject to the constraints of having *S* species, connectance *c* and a s.d. of interspecific interaction strength *σ*. The sub-matrices of **A** together with **M** are equivalent to May's matrices, but now we combine them with matrix **D** to form a meta-ecosystem. Elements of **D** are density-independent diffusion coefficients. Their arrangement determines the connectivity among localities. There are *n* patches (or ecosystems) and consequently the size of **J** is *n* × *S.* Our method of adding dispersal creates source–sink relations among the local communities in which net movement goes from high-density to low-density patches for each of the species involved.

The analysis of the random matrices **J** (Supplementary Note 1) gives us criteria for the stability threshold of meta-ecosystems. For the sake of simplicity, we consider a meta-ecosystem with global and homogenous dispersal, that is, a unique diffusion rate for all species between all pairs of patches (all the non-diagonal elements of **D** are equal to each other and all the diagonal elements of **D** are also equal; but see Supplementary Note 1 for analyses of more realistic cases) and we study partially heterogeneous patches. We allow the entries in the sub-matrices of **A**, *a*_*ij*_, to be correlated among the *n* local communities with correlation coefficient *ρ*; thus, *ρ* tends to 0 with increasing heterogeneity of *a*_*ij*_ among patches and to 1 when different patches exhibit the same values for *a*_*ij*_. In addition, as in May's approach, there is also no correlation among pairwise elements of the sub-matrices **A** (no specific predator–prey, competitive or mutualistic coupling). This case is the direct spatial extension to May's model and gives us a baseline for thinking about other possibilities. This last assumption is relaxed below with numerical simulations.

Analyses of random matrices such as those performed by May^3^,^4^ have been criticized for not considering the feasibility of the equilibrium (that is, such matrices may often give equilibria that have negative, and thus meaningless, densities for some of the species^28^). Previous work indicates that stability can be increased when the criterion is applied only to those communities that have positive values for all the species equilibrium densities^29^. In addition, from equation (2), dispersal does not directly enter the interaction sub-matrices **A** in the computation of the Jacobian. In reality however it should indirectly affect **A** through an effect on the equilibrium densities due to the role of emigration and immigration. We consequently conducted numerical simulations to fully capture the effect of dispersal on meta-ecosystem stability and account for the feasibility constraints. Our simulations started with the drawing of random interaction coefficients. We then solved the equilibrium for each matrix, in the absence of dispersal, and only retained those that gave with positive densities for all species. We subjected each such matrix to a gradient of dispersal and solved for the resulting largest eigenvalue numerically.

## Results

### Stability criterion for random matrices

The stability criteria for matrix **J** is generally complicated, but can be simplified under some conditions. Assuming that both *S* and *n* are large and that *d* is also sufficiently large, we obtain the following stability criterion (Fig. 2c):


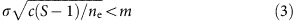


where 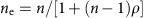 is the effective number of ecologically independent patches in the meta-ecosystem. When elements of the random matrices are perfectly correlated among patches (that is, *ρ=*1), equation (3) simplifies to May's formula, that is, equation (1). By contrasts, when elements of the random matrices are all completely independent (*ρ=*0), the stability criterion then reduces to 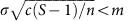, that is, the maximal admissible complexity parameter 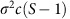
is multiplied by the number of patches.

The effect of the effective number of patches *n*_e_ in equation (3) results from the statistical thinning of the variance of the ‘average ecosystem' obtained by linking all ecosystems through dispersal (that is, as an instance of the central limit theorem). In other words, meta-ecosystems are stabilized because the main effect of dispersal is to make stability dependent on interaction coefficients averaged over many ecosystems, rather than on local interaction coefficients *per se*. By way of being average quantities, these meta-ecosystem interaction coefficients have lower variance, and hence correspond to an empirical spectral distribution with a smaller radius. These results indicate that dispersal can stabilize meta-ecosystem dynamics in proportion to its effective ecological size (the number of effectively independent habitat patches). To the degree that different local ecosystems have similar conditions (*ρ* increases and thus *n*_e_ decreases) however, this effect is weakened and the effect of dispersal disappears if all the local ecosystems are identical (that is, when *n*_e_=1 and *ρ=*1) and we recover May's equation (1).

When *d* is small (and *S* and *n* large), however, the effect of dispersal is very different (Supplementary Note 1 and Fig. 2b). The criterion corresponding to equation (3) in this situation is


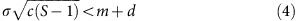


In this case, stability increases with *d*, regardless of the value of *n*_e_ and it does so additively (at least in cases where *d* is very small) in comparison with the criterion for isolated ecosystems. In this case, environmental heterogeneity is not important—emigration alone is the factor improving the stability of meta-ecosystems and its importance will depend on its magnitude relative to the intraspecific density dependence. Analytical results hold even in presence of heterogeneity among species in dispersal rates (Supplementary Note 1).

### Simulations of feasible meta-ecosystems

We were unable to find analytical solutions for intermediate levels of dispersal, so we turned to numerical simulations to do so. In addition, we used these simulations to address concerns that feasibility constraints (requiring non-negative equilibrium densities) might also affect our conclusions. An illustrative result for an average of 100 random meta-ecosystems of 15 species and 10 patches is shown in Fig. 3. We found that stability first increased with dispersal, peaking at intermediate rates and then slightly dropped and became almost insensitive to dispersal at high levels (Fig. 3a). Dispersal affects stability through its effects on various characteristics of the Jacobian matrix: the variance among its entries increased with the dispersal rate, the inter-patch correlation (*ρ*) increased and the mean diagonal elements decreased (Fig. 3b–d). Note that the inter-patch correlation coefficient increases but never reaches a value of 1 because, if biomass is more homogeneous with high dispersal, the per capita interaction coefficients would be independent of it and therefore the elements of **J** will never perfectly correlate. Finally, we also considered other topologies for dispersal and interactions to relax some of the assumptions of the random matrix theory. We find similar stability–dispersal relationships with local connectivity among localities (the extreme case of a spatially explicit dispersal; Fig. 4a). Further, simulations of more realistic predator–prey interaction matrices increases stability, but keep the relationship with dispersal.

## Discussion

Our analyses reveal that meta-ecosystem dynamics are stabilizing because of the effects of dispersal on the structure of the Jacobian matrix and its corresponding eigenvalues. In the case of high dispersal we find that the eigenvalues of the meta-ecosystem differ from those of local communities in two aspects. First, we find that a large subset of the real parts of eigenvalues of the community matrix are contained in a distribution with a range of 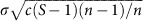, centred on a value of 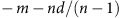, while the remaining *S* eigenvalues are enclosed in distribution of range 
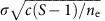
 centred around −*m* (Fig. 2). Dispersal thus affects stability through two mechanisms: (i) it moves the distribution of most of the eigenvalues towards more negative values; and (ii) it shrinks the range of the remaining eigenvalues in proportion to the number of effective patches (Fig. 2c). We call mechanism (i) the eigenvalue pushback effect and mechanism (ii) the Jacobian averaging effect. The mechanism involved at low dispersal differs (Fig. 2b). The primary effect of a low dispersal on the dynamics is emigration, which acts as a negative intraspecific feedback and is known to be stabilizing^30^. We call this mechanism (iii) the negative feedback effect.

The first part of our analysis follows May's formalism to facilitate the comparison with his work and comparisons with ongoing studies that use random matrix theory^16^. This approach allows us to rigorously identify the contribution of spatial dynamics to community stability by comparison with reference theory. We find that it reduces the effective complexity of the system (by shifting it from 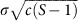 to 
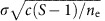
) and also promotes negative density dependence (it increases the diagonal elements). Analytical results (section 5 of Supplementary Note 1) also confirm this result is robust to variability of connectivity matrix (not all locations are connected to each other) and of diffusion coefficients (different species have different dispersal rates). The approximation of random matrix theory is a powerful analytical approach to provide a basis for comparison; however, it comes with several assumptions that have been raised repeatedly (for example, refs 9, 20), such as the random distribution of interaction strength, the shape of the distribution and the topology of the interactions. This approach has thus been criticized for its lack of realism, and the investigation of empirical ecosystems indeed revealed they contribute to affect the stability–complexity relationship^31^. Nevertheless, the basic qualitative results are still found in more complex scenarios even if they cannot be expressed in such simple ways. The approach thus has tremendous heuristic value even if it is not likely to adequately represent any real meta-ecosystems.

We nonetheless performed simulation analyses to relax some of the critical assumptions of random matrix theory and thereafter evaluate the robustness of our conclusions. Simulations account for the constraints that come from feasible systems, with different topologies and more realistic dispersal scenarios. Additional simulations confirmed our main conclusion that dispersal tends to stabilize meta-ecosystem dynamics. Even though these extra simulations are still idealized scenarios, they represent extreme cases with the reality somewhere between them and the assumption of random Jacobian matrices. Most of all, they confirm the generality of the mechanisms we underline, some of which had already been documented for simpler systems (see the review in ref. 25). Source–sink dynamics is a powerful stabilizing mechanism that arises as soon as there is some form of spatial heterogeneity and passive dispersal. It tends to homogenize the meta-ecosystem and thereby reduce the complexity of the Jacobian (the Jacobian averaging effect). While the assumption of diffusive dispersal could also be criticized, a density-dependent nonlinear dispersal process would even further stabilizes the dynamics because it would strengthen negative density dependence, moving the entire distribution of eigenvalues towards more negative values (the negative feedback effect).

Dispersal can promote local enrichment, compensatory dynamics and local coexistence because of source–sink dynamics^32^, and can consequently affect the feasibility as well as the stability of meta-ecosystems^33^. On the basis of simulation results (Fig. 3), we found that stability first increased with dispersal, peaked at intermediate rates and then slightly dropped and became almost insensitive to dispersal at high levels. The increase in stability agrees with both the eigenvalue pushback effect and the Jacobian averaging effect presented above. New mechanisms arise from the simulations because of the way we account for the feasibility constraint. First, we noted a slight decrease in the s.d. of the off diagonal elements of the Jacobian (Fig. 3b), although this effect might be negligible on average. We also found that on average, the correlation between patches of elements of the Jacobian does increase with dispersal (Fig. 3c). These two results could also be interpreted as a Jacobian averaging effect. Finally, we find that the diagonal elements of the Jacobian decrease linearly with dispersal (Fig. 3d), which correspond to the negative feedback effect. The maximization of stability at intermediate dispersal rates thus results from a mixture of the stabilizing and destabilizing effects of dispersal that we found in the limited cases described by our analytical approach.

A problem stemming from our simulations is the relatively high diagonal elements in the interaction matrix (intraspecific density dependence) in comparison to off-diagonal elements. This situation arises from the constraint of generating feasible communities from random interaction matrices. We are not the first to face this problem, for instance, Jansen and Kokkoris^34^ found that the probability of obtaining a feasible equilibrium decreases with connectance and mean interaction strength. Thébault and Fontaine^35^ also had to tune their diagonal elements to make sure they obtain feasible predator–prey and mutualistic communities. Similarly, Bastolla *et al*.^36^ considered strong intraspecific density dependence for plants when they investigated how the structure of mutualistic networks minimized competition and promoted coexistence. We know from the analytical theory that high diagonal elements only shift the distribution of eigenvalues (Fig. 2) but do not impact the relationship between stability and complexity. This conclusion, however, holds only if the strength of interactions, the diagonal elements and the diffusion coefficients are all independent of each other. If they do correlate (and they might, since the density dependence affects the equilibrium biomass and thus the elements of the Jacobian), then we might observe some discrepancy with the results we presented. For instance, a reduction in the diagonal elements is expected to increase the off-diagonal elements (switching from bottom-up to top-down regulation), which should decrease the stability. It is not clear however how this should interact with spatial dynamics, as very complex interactions would likely emerge.

Of course, May's approach, as well as ours, is highly abstract relative to what happens in natural communities. The new stabilizing mechanisms we reveal with our theoretical analysis nonetheless correspond to ecological processes that are well documented. The Jacobian averaging effect is essentially driven by source–sink dynamics. Exchanges of materials, energy and organisms have been widely documented for a whole range of ecosystems^32^,^37^ In general (with few exceptions), dispersal has tendency to homogenize systems with a redistribution of populations and abiotic material^32^. This coupling presumably stabilizes the dynamics^38^. The second mechanism, the negative feedback effect, is also evidently at play in many systems. In some cases, such as seed dispersal, the movement will not strongly affect the locality producing the propagules because their biomass is negligible relative to the source location. But for many organisms, emigration will negatively affect population dynamics in the source location and thus should act on its regulation. Territoriality is such an extreme case of density-dependent dispersal that should contribute to stabilize community dynamics. Although both mechanisms have been well documented in the field, what remains to test is their contribution to stability.

We found that spatial heterogeneity is key to the stabilizing effect of dispersal. Spatial heterogeneity on its own, in the absence of dispersal, increases the variability of interaction strength and should consequently reduce the stability. Heterogeneity, however, interacts with dispersal, via source–sink dynamics, and consequently the Jacobian averaging effect could only occur in spatially heterogeneous landscapes. Such heterogeneity could be driven by different primary productivities, or alternatively abiotic environmental conditions affecting the demography^39^ and the interaction strength^40^. Our analysis considers a relatively uniform meta-ecosystem, as species composition (presence/absence) remains essentially constant across the localities even if interaction coefficients and steady-state densities differed among them. Our results consequently do not directly apply to more contrasted meta-ecosystems, such as terrestrial–aquatic or above/below ground coupling. This distinction, however, does not prevent the mechanisms to play a role in such contexts; we even expect them to be reinforced in such situations. The Jacobian averaging effect will increase with the difference between localities. Similarly, the negative density dependence will be much stronger if there are no immigrants towards sources. Obviously further analysis will be required to assess the generality of our conclusions, but based on previous work and our understanding of the mechanisms at play, we expect them to be quite robust to the different assumptions of our models.

Additional stabilizing and destabilizing processes, such as the relative strengths of weak and strong feedbacks and frequency-dependent spatial effects^41^ or the proportions of the different types of interaction^16^, are also likely to be involved. A full understanding of their consequences, including the ways they may modify our general predictions for the stabilizing effects of dispersal in meta-ecosystem, is needed. Nevertheless, many plausible additions to the initial model proposed by May could actually increase stability even more in spatially structured ecosystems and thus act synergistically rather than antagonistically with the mechanisms we propose. For example, it has been shown that adaptive habitat selection by top predators can also be strongly stabilizing regardless of environmental heterogeneity^22^. In the same vein, adaptive foraging might be a strongly stabilizing factor in food webs^10^. Food webs may also be stabilized when top predators couple fast and slow energy channels^38^, for example, occurring in different patches of a meta-ecosystem, when interactions are structured as ‘weak links in long loops'^42^ or when the number of predator and prey species are (respectively) negatively and positively correlated with species body mass^14^. Many of these effects could be incorporated into a more detailed description of the matrix **J** and applying theory on the empirical spectral distribution of random matrices^17^.

We considered local stability analysis and Lotka–Volterra analysis to pursue the tradition initiated by May and to ensure our results are comparable to the long list of studies that followed. This approach has however strong limitations and is still distant from empirical investigations of stability. Among the most severe assumptions, it considers a linear approximation of the dynamics, in the neighbourhood of the equilibrium. Real systems are rarely at equilibrium, being constantly disturbed by environmental fluctuations and strong nonlinear interactions. Other metrics of stability should thus be considered, in addition to the asymptotic stability we studied here. For instance, recent analytical results on the reactivity of random matrices^43^,^44^ show that the dynamics during the transient phase following a disturbance could bring additional information, provided that the community matrix is not symmetric. Spatial dynamics could also be represented with strong fluctuations in densities^25^. Other measures of stability such as the spatial variance, the amplitude of fluctuations or persistence, could be considered as well. For instance, Gravel *et al*.^45^ found a positive relationship between the complexity of spatial food webs and their persistence when they are subject to colonization–extinction dynamics. Similarly, McCann *et al*.^22^ proposed that the capacity of a predator to switch between patches, fluctuating asynchronously, also reduces the magnitude of oscillations.

This discussion emphasizes that a major challenge for both theoretical and empirical ecologists is to integrate the multiple dimensions of stability. The contribution of spatial dynamics to stability of ecosystems is a multi-faceted problem that will only be solved by a diversity of approaches. The different metrics are not independent from one another and fortunately there are only a few dimensions to stability^46^. Future studies will require not only investigating these multiple dimensions but also the mechanisms linking them. Some of the metrics of stability, such as the coefficient of variation of population dynamics^47^, are easier to measure on the field than asymptotic stability. We consequently need to derive theory that would bridge the results from the investigation of random matrix theory and empirical measurements of stability.

Our study confirms that spatial dynamics in meta-ecosystems can be a remarkably strong stabilizing force that can facilitate coexistence among many interacting species. More importantly, our model indicates that this effect can be directly related to May's criteria and thus expands the applicability of random matrix theory to meta-ecosystems. Our model indicates that in cases with very low dispersal (low connectivity) the effects are proportional to the dispersal rate; that at high dispersal (high connectivity) the effects are proportional to the ecological size of the metacommunity, and that the effects are maximized at intermediate dispersal (intermediate connectivity). In relatively well-mixed meta-ecosystems, our results indicate that high species richness in local communities (*S*) can be easily explained if there is sufficient spatial heterogeneity in pairwise species interactions. Experimental work in natural systems indicates that species interactions do change with environmental context^48^,^49^ suggesting that simple spatial effects of dispersal could be a powerful stabilizing force that may explain very diverse communities of highly interactive species and thus provides a major possible solution to May's paradox of diversity. If so, high diversity is likely to depend critically on how landscapes are spatially structured by the joint effects of fragmentation (affecting dispersal) and the distribution of diverse local environments (affecting the ecological size of the meta-ecosystem).

## Methods

### Jacobian matrix structure

We used the following convention: all interaction and Jacobian matrices in our work are assumed to be ordered by patch (one matrix block=one patch), then species within a patch. For instance, the *S*th coefficient on the first row describes the effect of species *S* on species 1 in patch 1, while the *S*+1th coefficient describes the effect of species 1 from patch 2 on species 1 in patch 1.

### Random Jacobian model

We analysed the asymptotic empirical spectral distribution (ESD^17^) of Jacobian matrices following equation (2) when *S* goes towards infinity. Coefficients of the **A**_*x*_ matrices (intra-patch Jacobian within patch *x*) were obtained as follows: for each ordered pair of species *i*,*j*, an interaction exists with probability *c*. Because this applies to ordered pairs, this means that the action of *i* on *j* might exist while the reverse action do not. Each coefficient *a*_*ijx*_ is drawn from a normal distribution (mean=0 and s.d.=*σ*). In the homogenous case, the same is true except that all values of *a*_*ijx*_ are identical across all values of *x* (given *i* and *j*). The assumption of zero-mean is necessary, but normality is not^17^: as long as the distribution of **A**'s coefficients is independent and identically distributed, only the knowledge of its s.d. matters to uncover its asymptotic ESD.

The effects of the dispersal matrix **D** on the Jacobian depends on the homogeneity of dispersal among species. In the simple case, dispersal is assumed to be identical for all species, so that diagonal elements of intra-patch matrix blocks take value –*d* while inter-patch matrix blocks have *d*/(*n*–1) on the diagonal.

Further material on the ESD analysis, as well as results on more complex cases incorporating non-global and heterogeneous dispersal, is provided in Supplementary Information.

### Numerical analysis

We considered a general implementation of a Lotka–Volterra model with spatial dynamics. The model represents the dynamics of species *i* at location *x* given its interaction with all *S* species *j* and *n* locations *y*:


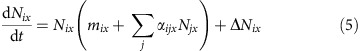


where *m*_*ix*_ is the intrinsic rate of increase, *N*_*ix*_ is the density of species *i* at location *x*, *α*_*ijx*_ is the per capita effect of species *j* on species *i* at location *x* (which is not necessary symmetric) and Δ*N*_*ix*_ is the migration balance for species *i* at location *x*. The latter could be based on several functional forms for dispersal (for example, dispersal, density-dependent dispersal and so on). Here we considered passive dispersal and used a general function allowing different rates across locations and species:


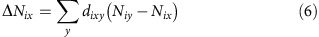


where *d*_*ixy*_ is the dispersal rate of species *i* between locations *x* and *y*. Each species therefore exchanges individuals between locations *x* and *y* at rate *d*_*ixy=*_*d*_*iyx*_*=d/*(*n*–1). When simulating Lotka–Volterra dynamics, we only considered the simplest system where all localities are connected to each other with the same rate for all species and localities.

### Parameterization

We follow the rules used by May^3^,^4^ to facilitate the comparison. An interaction occurs with probability *c=*0.2. Interspecific interaction coefficients are drawn randomly from a normal distribution centred on zero and with a s.d. of 0.25. We also considered a predator–prey structure determined according to iterations of the niche model of food web structure^50^. For this particular case, we specified that the interaction coefficients from a prey to a predator has the same absolute value but opposed sign to the interaction coefficient from predator to the prey. All diagonal elements (intraspecific interaction coefficients) are set to –1. A regional average interaction coefficient is drawn for all pairs of species and then this coefficient is multiplied by a random normal deviate with mean of 1 and s.d. of 0.1 to account for spatial environmental heterogeneity (thus generating a positive correlation between inter-patch elements). Only the non-null elements of the interaction matrix are affected by environmental variability.

**Figure 1:**
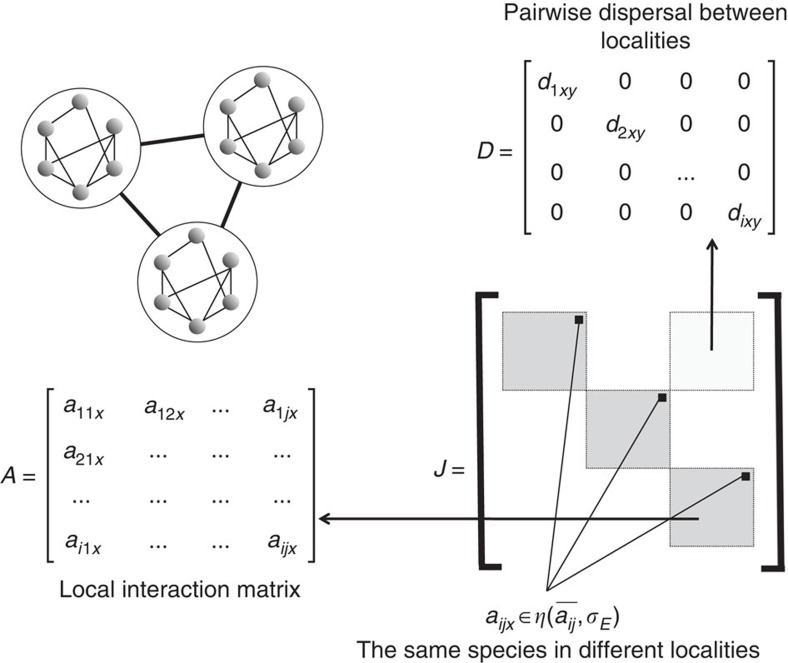
Conceptual illustration of the Jacobian matrix in random meta-ecosystems. The model represents the dynamics of a meta-ecosystem, pictured as a spatial network of interaction networks (top left). The Jacobian matrix representing all interactions among pairs of species and locations is highly structured. It is made of the sub-matrices **A**, **M** and **D**. The spatial heterogeneity among locations is implemented by varying interaction coefficients in space (different entries in each interaction sub-matrix) and the landscape is implemented by varying the spatial structure of the model (entries in each pairwise dispersal matrix).

**Figure 2:**
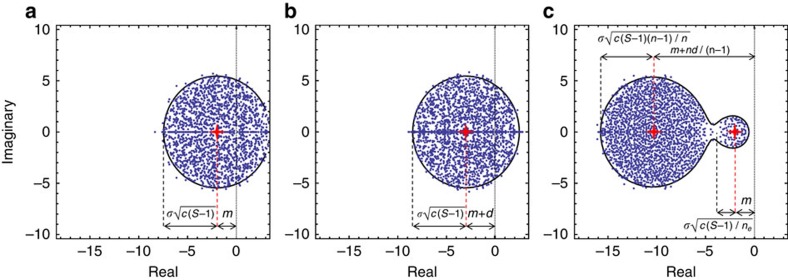
Illustration of the distribution of eigenvalues in the complex plane. Distribution of eigenvalues of matrix **J** in the complex plane when *σ*=1, *n*_e_=*n*=20, *S*=100, *c*=0.3, *m*=2, and (**a**) *d*=0, (**b**) *d*=1 and (**c**) *d*=8. Solid lines indicate predictions from asymptotic distributions of eigenvalues under the circle law^16^; individual points indicate eigenvalues obtained from a random drawing of individual Jacobian matrices following a Gaussian distribution for non-diagonal elements. Red stars indicate the position of the centres of eigenvalue distributions following (**b**) the negative feedback effect for small values of dispersal and (**c**) the eigenvalue pushback effect for large values of diffusion.

**Figure 3:**
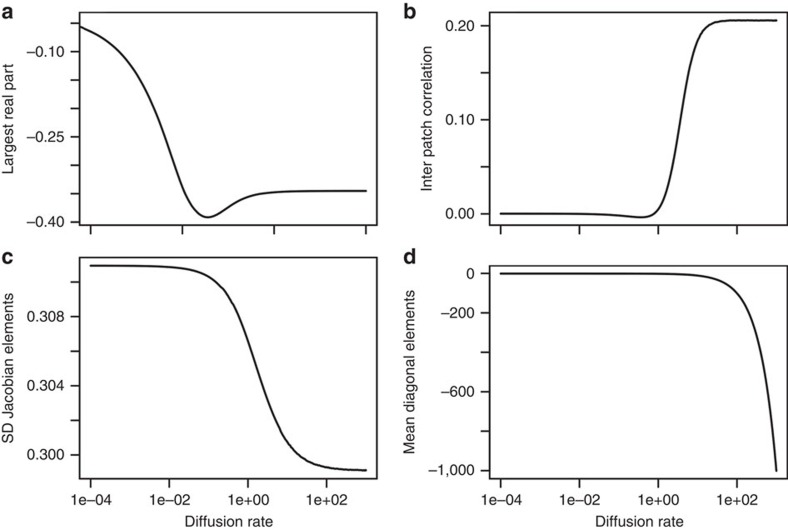
Effect of diffusion rate on Jacobian matrices and their stability. (**a**) The real part of the largest eigenvalue, (**b**) the s.d. of non-null elements of the Jacobian matrix, (**c**) inter-patch correlation and (**d**) mean of the diagonal elements. Meta-ecosystems were generated randomly with the constraint that all populations have positive equilibrium densities. Each line represents the average of 100 replicated random meta-ecosystems with increasing diffusion rate. See the Supplementary Information for parameters.

**Figure 4:**
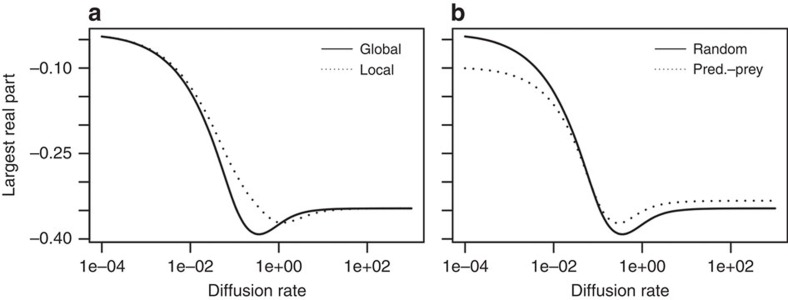
Effect of different topologies on the effect of dispersal on stability. (**a**) Comparison between global dispersal and local dispersal (nearest neighbour). (**b**) Comparison between random interaction topologies (as in May^3^,^4^) and predator–prey topologies (as in Williams and Martinez^50^). Each line represents the average of 100 replicated random meta-ecosystems with increasing diffusion.

The definition of the model (Fig. 1) is general enough to account for all types of spatial variation of the environment. As with the work on Jacobian matrices, we considered the extreme scenario of covariance among species in their response to environmental variability where the random deviation from the average pairwise interaction *α*_*ijx*_ is independent among all species. The analytical results suggest that the results should be similar whether only off diagonal elements or the diagonal elements are subject to environmental variation and we consequently only considered the former for simulations. All simulations are conducted assuming a global dispersal (all patches are related to each other by equal diffusion rates) except when specified otherwise. Additional simulations were run with nearest local connectivity (patch *x* being connected to patch *x*–1 and *x+*1). A torus shape was assumed so that the patch *x*=1 is connected to patch *x*=2 and *x*=10.

We investigated the impact of increasing dispersal on the stability of feasible random meta-ecosystems. To do so, we ran simulations on a gradient of dispersal values, *d*, ranging from 0.001 to 1,000, with a step of 0.01 on a logarithmic scale. We drew random interaction matrices according to the above rules. We then combined interaction matrices with dispersal and followed the standard procedure of local stability analysis with the Lotka–Volterra system of equations: calculation of the equilibrium solution to the system of equation, linearization of the system of equations around the equilibrium to obtain the Jacobian matrix and finally computation of the eigenvalues using numerical methods. All simulations were run with *S*=15 species and *N*=10 locations. We present results for 1,000 replicates of random meta-ecosystems for each parameter combination.

### Data availability

The authors declare that all data supporting the findings of these studies are available with the article and its Supplementary Information File.

## Additional information

**How to cite this article:** Gravel, D. *et al*. Stability and complexity in model meta-ecosystems. *Nat. Commun.* 7:12457 doi: 10.1038/ncomms12457 (2016).

## Supplementary Material

Supplementary Information Supplementary Note 1, Supplementary References
