# Supplementary material for: Stability and complexity in model meta-ecosystems
Source: Nat Commun. 2016 Aug 24;7:12457. doi: 10.1038/ncomms12457 (PMC4999499; doi:10.1038/ncomms12457)
Supplement: Supplementary Information — Supplementary Note 1, Supplementary References [file ncomms12457-s1.pdf]

Supplementary Notes

Supplementary Note 1

In this appendix, we are interested in determining the empirical spectral distribution (ESD) of the Jacobian matrix of a metaecosystem model. In the first section, we derive the support of the ESD for a Jacobian matrix in which within-patch Jacobians are independent and dispersal is homogeneous (the "heterogeneous Jacobian/homogeneous dispersal" case). In the second section, we tackle the opposite case where all within-patch Jacobians are equal (the "homogeneous Jacobian/homogeneous dispersal" case). In the third section, we come back to the heterogenous Jacobian case and apply a basis change inherited from the second section to obtain an alternate proof. In the fourth section, we use this alternate proof to tackle the case where the Jacobian matrix is a mix of homogeneous and heterogeneous matrices. In the fifth section, we examine the case of heterogenous Jacobian matrices with only a limited number of neighbouring patches.

In brief, stabilization through dispersal is manifested through three different effects. The first spatial effect on stability ("*eigenvalue pushback effect*") comes from inequality (1.6), i.e. the fact that the limit support for the ESD of the Jacobian matrix will consist in a small disk around $-m$ and a large disk around $-m - \frac{nd}{n-1}$. The second spatial effect ("*Jacobian averaging effect*"), highlighted in sections 1, 3 and 4, simply consists in the fact that, when within-patch Jacobian matrices are heterogeneous (not perfectly correlated, see inequality [4.22]), any non-zero dispersal will thin the effective variance of the Jacobian coefficients (through a phenomenon akin to the central limit theorem) and, thus, make the stability criterion less stringent. The third spatial effect ("*negative feedback effect*") corresponds to the raw stabilizing effect of emigration at low dispersal rates (equations [1.8,4.31]; when dispersal is weak, its principal effect is to act as a supplementary negative feedback on populations producing emigrants).

Preamble: finding the support of the ESD for random matrices

Here, we describe the method inherited from the results of Tao et al.[3] to compute the support of the ESD of matrix $\mathbf{X} = \mathbf{A} + \mathbf{B}$ of size n, with $\mathbf{A}$ being a random matrix (all its elements are i.i.d. drawings from a distribution with mean 0 and variance σ^2) and $\mathbf{B}$ is a deterministic matrix with eigenvalues noted $\{b_1, ..., b_n\}$. For $\sigma = 1$, the support of $\mathbf{X}/\sqrt{n}$ converges when $n \to \infty$ towards the set of complex points z that obey:

$$\int \frac{\mu_{B/\sqrt{n}}(u)}{|z-u|^2} \geq 1 \tag{0.1}$$

where $\mu_{B/\sqrt{n}}$ is the measure of $\{b_1/\sqrt{n}, ..., b_n/\sqrt{n}\}$, i.e. using Dirac deltas:

$$\mu_{B/\sqrt{n}}(u) = \frac{1}{n} \sum_{k=1}^{n} \delta_{b_k/\sqrt{n}}(u) du \tag{0.2}$$

When $\sigma \neq 1$, finding the support of $\mathbf{X}/\sigma\sqrt{n}$ is still given by:

$$\int \frac{\mu_{B/\sigma\sqrt{n}}(u)}{|z-u|^2} \geq 1 \tag{0.3}$$

or using the set of eigenvalues of $\mathbf{B}$:

$$\frac{1}{n} \sum_{k=1}^{n} \frac{1}{\left|z - \frac{b_k}{\sigma\sqrt{n}}\right|^2} \geq 1 \tag{0.4}$$

The "stability criterion", i.e. assuming that $\mathbf{X}$ represents a Jacobian matrix, the condition that puts the support of its ESD out of $\mathbb{R}^+$, is given by:

$$\max_z \left[\sigma\sqrt{n} \times \Re(z) \, when \, \frac{1}{n} \sum_{k=1}^{n} \frac{1}{\left|z - \frac{b_k}{\sigma\sqrt{n}}\right|^2} \geq 1 \right] < 0 \tag{0.5}$$

Using $y = \sigma\sqrt{n}z$, this criterion is also:

$$\max_y \left[\Re(y)\, when \sum_{k=1}^{n} \frac{1}{|y-b_k|^2} \geq \frac{1}{\sigma^2} \right] < 0 \tag{0.6}$$

As an example, if all b_k are equal to b, the maximum real admissible y is $b + \sigma\sqrt{n}$, so that the stability criterion becomes:

$$\sigma\sqrt{n} < -b \tag{0.7}$$

1 Heterogeneous Jacobian and homogeneous dispersal

Consider the following Jacobian matrix:

$$\mathbf{J} = -\mathbf{M} + \mathbf{D} + \mathbf{A} \tag{1.1}$$

where $\mathbf{M}$ is the diagonal matrix with value m on the diagonal and 0 in the rest of the matrix; $\mathbf{D}$ is the matrix representing the effect of dispersal among patches; and $\mathbf{A}$ is the collection of Jacobian matrices, arranged as diagonal blocks, which describe the Jacobian matrices that would have arised in isolated communities (except for diagonal terms which are contained in the term $-\mathbf{M}$). Consecutive blocks of size S describe the stability properties within patches, and the total size of all matrices is $n \times S$ where n is the number of patches.

We will assume that the effect of dispersal is diffusive and homogeneous (with diffusion parameter d), so that that $\mathbf{D}$ can be written as:

$$\mathbf{D} = \begin{pmatrix} -d\mathbf{I}_S & \frac{d}{n-1}\mathbf{I}_S & ... & \frac{d}{n-1}\mathbf{I}_S \\ \frac{d}{n-1}\mathbf{I}_S & -d\mathbf{I}_S & ... & \frac{d}{n-1}\mathbf{I}_S \\ ... & ... & ... & ... \\ \frac{d}{n-1}\mathbf{I}_S & \frac{d}{n-1}\mathbf{I}_S & ... & -d\mathbf{I}_S \end{pmatrix} \tag{1.2}$$

where $\mathbf{I}_S$ is the identity matrix of size S.

We will also assume that the effects of within-patch interactions on the Jacobian are heterogeneous, i.e. that $\mathbf{A}$ can be written as:

$$\mathbf{A} = \begin{pmatrix} \mathbf{A}_1 & \mathbf{0} & ... & \mathbf{0} \\ \mathbf{0} & \mathbf{A}_2 & ... & \mathbf{0} \\ ... & ... & ... & ... \\ \mathbf{0} & \mathbf{0} & ... & \mathbf{A}_n \end{pmatrix} \tag{1.3}$$

where random matrices $\mathbf{A}_k$ of size S (with elements a_{ijk}) are independent and follow:

$$\begin{aligned} a_{iik} &= 0 \\ a_{ijk} &\sim \mathcal{B}(c) \times \mathcal{N}\left(0, \sigma^2\right) \end{aligned} \tag{1.4}$$

In other words, non-diagonal elements of the $\mathbf{A}_k$ matrices follow Gaussian distributions with probability c, and are equal to 0 with probability $1-c$. Note that the variance $\mathbb{V}\left[\mathbf{A}_k\right]$ of $\mathbf{A}_k$ entries is equal to $\left(1 - \frac{1}{S}\right) c\sigma^2$. The normality assumption is, strictly speaking, not needed[3] - only finite variance and zero means suffice to apply the circular law (see preamble above).

Applying the circular law to $-m\mathbf{I}_S + \mathbf{A}_1$, the corresponding stability criterion for large S is given by inequality (0.7) with the appropriate values for parameters b, n and σ[2,1]:

$$\sigma\sqrt{c\left(1 - \frac{1}{S}\right)} \times \sqrt{S} < m \tag{1.5}$$

In the absence of dispersal ($d = 0$), the asymptotic (for large S) stability criterion of $\mathbf{J} = -\mathbf{M} + \mathbf{A}$ is also given by inequality (1.5) because the new matrix size is of size nS and the new connectance is nc/n^2 (only n out of the n^2 blocks have non-zero entries - this entails that the new variance is $1/n$ times the one we had with non-spatially structured Jacobians). This is also understandable as follows: each matrix $\mathbf{A}_k - m\mathbf{I}_S$ contributes with its own eigenvalues to the ESD of $\mathbf{J}$; since the $\mathbf{A}_k - m\mathbf{I}_S$ are independent and have the same ESD support, the limiting distribution of the ESD support of $\mathbf{J}$ is also the same.

With dispersal ($d > 0$), the deterministic part of $\mathbf{J}$ becomes $\mathbf{D} - \mathbf{M}$, which has two eigenvalues, $-m$ and $-m - \frac{nd}{n-1}$, with respective multiplicities S and $(n-1)S$. In this case, the limit support of the ESD of $\mathbf{D} - \mathbf{M} + \mathbf{A}$ becomes (equations [0.3] and [0.6]):

$$\frac{S}{|z+m|^2} + \frac{(n-1)S}{\left|z+m+\frac{nd}{n-1}\right|^2} \geq \frac{n}{\sigma^2 c\left(1-\frac{1}{S}\right)} \tag{1.6}$$

For sufficiently large d, this limit support resembles a set of two disks, one centered at $-m$, with S eigenvalues, and another one centered at $-m - \frac{nd}{n-1}$, with $(n-1)S$ eigenvalues. However, contrary to the previous case in which the radius of the limit disk did not vary with n, here the "radius" of the disk centered at $-m$ is $\sigma\sqrt{c(S-1)/n}$, i.e. decreases with increasing n. In ecological terms, this means that more habitat patches with heterogeneous Jacobian matrices are stabilizing for sufficiently large d. In statistical terms, the variance associated with the first disk center decreases as $1/n$, following the central limit theorem. When d is large, the maximum admissible real z for inequality (1.6) is (at first available order in $1/d$) $z^* = -m + \sigma\sqrt{c(S-1)/n} + (n-1)^3\left[\sigma^2 c(S-1)\right]^{3/2}/2n^3d^2$, so that the stability criterion is:

$$\frac{\sigma\sqrt{c(S-1)}}{\sqrt{n}}\left[1 + \frac{(n-1)^3\sigma^2 c(S-1)}{2d^2n^3}\right] < m \tag{1.7}$$

For very small d, on the other hand, all eigenvalues are clustered around $-m$, and the first order (in d) stability criterion becomes:

$$\sigma\sqrt{c(S-1)} < m + d \tag{1.8}$$

This stabilizing effect of dispersal corresponds to the "*negative feedback effect*" mentioned in the main text.

2 Homogeneous Jacobian and homogeneous dispersal

We now consider the Jacobian matrix $\mathbf{J}$ as in equation (1.1) with homogeneous within-patch Jacobian matrices, i.e. such that:

$$\mathbf{A} = \begin{pmatrix} \mathbf{A}_0 & \mathbf{0} & ... & \mathbf{0} \\ \mathbf{0} & \mathbf{A}_0 & ... & \mathbf{0} \\ ... & ... & ... & ... \\ \mathbf{0} & \mathbf{0} & ... & \mathbf{A}_0 \end{pmatrix} \tag{2.1}$$

Clearly, the limiting distribution of the ESD of $\mathbf{A}$ is equal to that of $\mathbf{A}_0$, with each eigenvalue having multiplicity n. In the absence of dispersal, the limit ESD support for $\mathbf{J}$ is not different from the one found for heterogeneous Jacobians (i.e. a disk centered at $-m$ and of radius $\sigma\sqrt{c(S-1)}$), but the limit ESD is different in that it converges towards the whole disk with S, while it did with nS (and hence more rapidly) in the heterogeneous case. Esssentially, convergence towards the whole disk is slowed because each eigenvalue has multiplicity n.

The matrix $\mathbf{D} - \mathbf{M}$ is easy to diagonalize. Indeed, $\mathbf{P}^{-1}(\mathbf{D} - \mathbf{M})\mathbf{P}$ is diagonal, with the S first eigenvalues equal to $-m$, when

$$\mathbf{P} = \begin{pmatrix} \mathbf{I}_S & -\mathbf{I}_S & -\mathbf{I}_S & ... & -\mathbf{I}_S \\ \mathbf{I}_S & \mathbf{I}_S & \mathbf{0} & ... & \mathbf{0} \\ \mathbf{I}_S & \mathbf{0} & \mathbf{I}_S & ... & \mathbf{0} \\ ... & ... & ... & ... & ... \\ \mathbf{I}_S & \mathbf{0} & \mathbf{0} & ... & \mathbf{I}_S \end{pmatrix} \tag{2.2}$$

and

$$\mathbf{P}^{-1} = \frac{1}{n}\begin{pmatrix} \mathbf{I}_S & \mathbf{I}_S & \mathbf{I}_S & ... & \mathbf{I}_S \\ -\mathbf{I}_S & (n-1)\mathbf{I}_S & -\mathbf{I}_\mathbf{S} & ... & -\mathbf{I}_\mathbf{S} \\ -\mathbf{I}_S & -\mathbf{I}_\mathbf{S} & (n-1)\mathbf{I}_S & ... & -\mathbf{I}_\mathbf{S} \\ ... & ... & ... & ... & ... \\ -\mathbf{I}_S & -\mathbf{I}_S & -\mathbf{I}_S & ... & (n-1)\mathbf{I}_S \end{pmatrix} \tag{2.3}$$

It is easy to compute that

$$\mathbf{P}^{-1}\mathbf{A}\mathbf{P} = \mathbf{A} \tag{2.4}$$

so that the following relation holds:

$$\mathbf{P}^{-1}\mathbf{J}\mathbf{P} = \begin{pmatrix} \mathbf{A}_0 - m\mathbf{I}_S & \mathbf{0} & \mathbf{0} & ... & \mathbf{0} \\ \mathbf{0} & \mathbf{A}_0 - (m + \frac{nd}{n-1})\mathbf{I}_S & \mathbf{0} & ... & \mathbf{0} \\ \mathbf{0} & \mathbf{0} & \mathbf{A}_0 - (m + \frac{nd}{n-1})\mathbf{I}_S & ... & \mathbf{0} \\ ... & ... & ... & ... & ... \\ \mathbf{0} & \mathbf{0} & \mathbf{0} & ... & \mathbf{A}_0 - (m + \frac{nd}{n-1})\mathbf{I}_S \end{pmatrix} \tag{2.5}$$

Because the ESDs of $\mathbf{J}$ and $\mathbf{P}^{-1}\mathbf{J}\mathbf{P}$ are equal, we conclude that the limit ESD of $\mathbf{J}$ consists in S times the limit ESD of $\mathbf{A}_0 - m\mathbf{I}_S$ and $(n-1)S$ times the limit ESD of $\mathbf{A} - (m + \frac{nd}{n-1})\mathbf{I}_S$. For sufficiently large d, the condition for stability is the same as given by equation (1.5), i.e.

$$\sigma\sqrt{c(S-1)} < m \tag{2.6}$$

.

3 Revisiting the case of the heterogeneous Jacobian

Taking the case where $\mathbf{A}$ is defined by equation (1.3), the transformation applied in equation (2.4) yields:

$$\mathbf{P}^{-1}\mathbf{A}\mathbf{P} = \begin{pmatrix} \overline{\mathbf{A}} & \frac{1}{n}(\mathbf{A}_2 - \mathbf{A}_1) & \frac{1}{n}(\mathbf{A}_3 - \mathbf{A}_1) & ... & \frac{1}{n}(\mathbf{A}_n - \mathbf{A}_1) \\ \mathbf{A}_2 - \overline{\mathbf{A}} & \mathbf{A}_2 + \frac{1}{n}(\mathbf{A}_1 - \mathbf{A}_2) & \frac{1}{n}(\mathbf{A}_1 - \mathbf{A}_3) & ... & \frac{1}{n}(\mathbf{A}_1 - \mathbf{A}_n) \\ \mathbf{A}_3 - \overline{\mathbf{A}} & \frac{1}{n}(\mathbf{A}_1 - \mathbf{A}_2) & \mathbf{A}_3 + \frac{1}{n}(\mathbf{A}_1 - \mathbf{A}_3) & ... & \frac{1}{n}(\mathbf{A}_1 - \mathbf{A}_n) \\ ... & ... & ... & ... & ... \\ \mathbf{A}_n - \overline{\mathbf{A}} & \frac{1}{n}(\mathbf{A}_1 - \mathbf{A}_2) & \frac{1}{n}(\mathbf{A}_1 - \mathbf{A}_3) & ... & \mathbf{A}_n + \frac{1}{n}(\mathbf{A}_1 - \mathbf{A}_n) \end{pmatrix} \tag{3.1}$$

where $\overline{\mathbf{A}} = \frac{1}{n}\sum_i \mathbf{A}_i$. When n is large, all matrix blocks in equation (3.1) that have variance of order $o\left(\frac{1}{n^2}\right)$ can be removed (remember that $\mathbb{V}\left[\overline{\mathbf{A}}\right]$ is of order $\frac{1}{n}$), so that:

$$\mathbf{P}^{-1}\mathbf{A}\mathbf{P} \approx \begin{pmatrix} \overline{\mathbf{A}} & \mathbf{0} & \mathbf{0} & ... & \mathbf{0} \\ \mathbf{A}_2 - \overline{\mathbf{A}} & \mathbf{A}_2 + \frac{1}{n}(\mathbf{A}_1 - \mathbf{A}_2) & \mathbf{0} & ... & \mathbf{0} \\ \mathbf{A}_3 - \overline{\mathbf{A}} & \mathbf{0} & \mathbf{A}_3 + \frac{1}{n}(\mathbf{A}_1 - \mathbf{A}_3) & ... & \mathbf{0} \\ ... & ... & ... & ... & ... \\ \mathbf{A}_n - \overline{\mathbf{A}} & \mathbf{0} & \mathbf{0} & ... & \mathbf{A}_n + \frac{1}{n}(\mathbf{A}_1 - \mathbf{A}_n) \end{pmatrix} \tag{3.2}$$

When n is suffciently large, we thus observe that $\mathbf{P}^{-1}\mathbf{J}\mathbf{P}$ is approximately:

$$\mathbf{P}^{-1}\mathbf{J}\mathbf{P} \approx \begin{pmatrix} \overline{\mathbf{A}} - m\mathbf{I}_S & \mathbf{0} & ... & \mathbf{0} \\ \mathbf{A}_2 - \overline{\mathbf{A}} & \mathbf{A}_2 + \frac{1}{n}(\mathbf{A}_1 - \mathbf{A}_2) - (m + \frac{nd}{n-1})\mathbf{I}_S & ... & \mathbf{0} \\ \mathbf{A}_3 - \overline{\mathbf{A}} & \mathbf{0} & ... & \mathbf{0} \\ ... & ... & ... & ... \\ \mathbf{A}_n - \overline{\mathbf{A}} & \mathbf{0} & ... & \mathbf{A}_n + \frac{1}{n}(\mathbf{A}_1 - \mathbf{A}_n) - (m + \frac{nd}{n-1})\mathbf{I}_S \end{pmatrix} \tag{3.3}$$

Because the ESD of a block triangular matrix is the union of the ESDs of its diagonal blocks, the asymptotic stability criterion $\sigma\sqrt{c(S-1)/n} < m$ (limit when $d \to \infty$ of inequality 1.7) results from the fact that $\mathbb{V}\left[\overline{\mathbf{A}}\right] = \mathbb{V}\left[\mathbf{A}\right]/n$. However, this proof is weaker than the one given in section 1 because it relies on large n values.

4 Mixed Jacobian with homogeneous dispersal

We now assume that matrix $\mathbf{A}$ has elements a_{ijk} defined by:

$$\begin{aligned} a_{iik} &= 0 \\ a_{ijk} &\sim \mathcal{B}(c) \times [\alpha_{ij} + \beta_{ijk}] \\ \alpha_{ij} &\sim \mathcal{N}\left(0, \sigma_0^2\right) \\ \beta_{ijk} &\sim \mathcal{N}\left(0, \sigma_E^2\right) \end{aligned} \tag{4.1}$$

In other words, each non-diagonal entry of $\mathbf{A}$ is zero with probability $1-c$ or, with probability c, is equal to the sum of two Gaussian distributions, one common to all patches (the α term) and one idiosyncratic to each species and patch (the β term). In matrix notation, we will note:

$$\mathbf{A} = \begin{pmatrix} \mathbf{A}_0 + \mathbf{A}_1 & \mathbf{0} & ... & \mathbf{0} \\ \mathbf{0} & \mathbf{A}_0 + \mathbf{A}_2 & ... & \mathbf{0} \\ ... & ... & ... & ... \\ \mathbf{0} & \mathbf{0} & ... & \mathbf{A}_0 + \mathbf{A}_n \end{pmatrix} \tag{4.2}$$

where $\mathbf{A}_0$ corresponds to the α terms in equation (4.1).

4.1 Stability criterion obtained at large d and large n

From equation (2.4) and (3.2) applied, respectively, to $\mathbf{A}_0$ and the set of blocks $\{\mathbf{A}_1, \mathbf{A}_2, ..., \mathbf{A}_n\}$, and taking large n and d values, we obtain the following stability criterion:

$$\sqrt{c(S-1)\left[\sigma_0^2 + \frac{\sigma_E^2}{n}\right]} < m \tag{4.3}$$

4.2 Stability criterion at large d

Another way of looking at the eigenvalues of $\mathbf{J}$ when $\mathbf{A}$ is defined by equation (4.3) is to consider $\mathbf{A}_0$ as belonging to the "deterministic part" of $\mathbf{J}$. If $\{\lambda_1, \lambda_2, ..., \lambda_S\}$ refer to the eigenvalues of $\mathbf{A}_0$ and we also write $\mathbf{A}_0$ for the $nS \times nS$ matrix that has $\mathbf{A}_0$ as diagonal blocks (and 0 everywhere else), the support of the ESD of $\mathbf{D} - \mathbf{M} + \mathbf{A}_0 + (\mathbf{A} - \mathbf{A}_0) / \sigma_E \sqrt{c(S-1)}$ is given by:

$$\frac{1}{nS}\sum_{k=1}^{S} \frac{1}{|z + m - \lambda_k|^2} + \frac{n-1}{nS}\sum_{k=1}^{S} \frac{1}{\left|z + m + \frac{nd}{n-1} - \lambda_k\right|^2} \geq \frac{1}{\sigma_E^2 c(S-1)} \tag{4.4}$$

When $d \to \infty$, the approximation of the left-hand side of inequality (4.4) at the first available order in $1/d$ yields:

$$\frac{1}{nS}\sum_{k=1}^{S} \frac{1}{|z + m - \lambda_k|^2} + \frac{1}{d^2}\left(\frac{n-1}{n}\right)^3 \geq \frac{1}{\sigma_E^2 c(S-1)} \tag{4.5}$$

4.2.1 Conservative criterion

From inequality (4.5), we can obtain a conservative estimate of the stability criterion by virtually allowing all λ's to concentrate at the value with the highest real part (call it λ^*). Inequality (4.5) thus becomes:

$$\frac{1}{n}\frac{1}{|z + m - \lambda^*|^2} + \frac{1}{d^2}\left(\frac{n-1}{n}\right)^3 \geq \frac{1}{\sigma_E^2 c(S-1)} \tag{4.6}$$

which yields the following stability criterion (analogous to inequality [1.7]):

$$\frac{\sigma_E \sqrt{c(S-1)}}{\sqrt{n}}\left[1 + \frac{(n-1)^3 \sigma_E^2 c(S-1)}{2d^2 n^3}\right] < \sqrt{[m - \Re(\lambda^*)]^2 + \Im(\lambda^*)^2} \tag{4.7}$$

The support of the ESD of $\mathbf{A}_0$ is such that its eigenvalue with highest real part always obeys:

$$\Re(\lambda^*) \leq \sigma_0 \sqrt{c(S-1)} \tag{4.8}$$

Plugging inequality (4.8) into inequality (4.7), assuming $\Im(\lambda^*) = 0$ and taking the left-hand side of the inequality to its maximum value, we obtain the following (conservative) stability criterion:

$$\frac{\sigma_E \sqrt{c(S-1)}}{\sqrt{n}}\left[1 + \frac{(n-1)^3 \sigma_E^2 c(S-1)}{2d^2 n^3}\right] + \sigma_0 \sqrt{c(S-1)} < m \tag{4.9}$$

When taking only members of order zero in $1/d$, this leads to inequality (4.3).

4.2.2 Asymptotically exact criterion

Taking the limit of a very large S in inequality (4.4), one can transform the finite sum over λ_k into an integral over the uniform distribution on the disk of radius $\sigma_0\sqrt{c(S-1)}$:

$$\frac{1}{n\pi\sigma_0^2 c(S-1)}\int_{|\lambda|\leq\sigma_0\sqrt{c(S-1)}}\frac{d\lambda}{|z+m-\lambda|^2}+\frac{n-1}{n\pi\sigma_0^2 c(S-1)}\int_{|\lambda|\leq\sigma_0\sqrt{c(S-1)}}\frac{d\lambda}{\left|z+m+\frac{nd}{n-1}-\lambda\right|^2}\geq\frac{1}{\sigma_E^2 c(S-1)} \tag{4.10}$$

With $d\lambda = rdrd\theta$ as the natural measure of surface for the disk, the first integral becomes:

$$\int_{|\lambda|\leq\sigma_0\sqrt{c(S-1)}}\frac{d\lambda}{|z+m-\lambda|^2}=\int_{r=0}^{\sigma_0\sqrt{c(S-1)}}\int_{\theta=0}^{2\pi}\frac{rdrd\theta}{|z+m-re^{i\theta}|^2} \tag{4.11}$$

Given the value of the integral when $z\in\mathbb{R}^-$ and $z>-m+\sigma_0\sqrt{c(S-1)}$:

$$\int_{|\lambda|\leq\sigma_0\sqrt{c(S-1)}}\frac{d\lambda}{|z+m-\lambda|^2}=\pi\log\left[\frac{(m+z)^2}{\left[m+z+\sigma_0\sqrt{c(S-1)}\right]\left[m+z-\sigma_0\sqrt{c(S-1)}\right]}\right] \tag{4.12}$$

The same result can be obtained for the second integral:

$$\int_{|\lambda|\leq\sigma_0\sqrt{c(S-1)}}\frac{d\lambda}{\left|z+m+\frac{nd}{n-1}-\lambda\right|^2}=\pi\log\left[\frac{\left(m+\frac{nd}{n-1}+z\right)^2}{\left[m+\frac{nd}{n-1}+z+\sigma_0\sqrt{c(S-1)}\right]\left[m+\frac{nd}{n-1}+z-\sigma_0\sqrt{c(S-1)}\right]}\right] \tag{4.13}$$

Hence, the admissibility criterion for z is given by:

$$\begin{array}{r}\log\left(\frac{(m+z)^2}{\left[m+z+\sigma_0\sqrt{c(S-1)}\right]\left[m+z-\sigma_0\sqrt{c(S-1)}\right]}\right)\\+(n-1)\log\left(\frac{\left(m+\frac{nd}{n-1}+z\right)^2}{\left[m+\frac{nd}{n-1}+z+\sigma_0\sqrt{c(S-1)}\right]\left[m+\frac{nd}{n-1}+z-\sigma_0\sqrt{c(S-1)}\right]}\right)\end{array}\geq\frac{n\sigma_0^2}{\sigma_E^2} \tag{4.14}$$

The corresponding stability criterion, at first available order in $1/d$, is thus given by:

$$\frac{\sigma_0\sqrt{c(S-1)}e^{\frac{n\sigma_0^2}{4\sigma_E^2}}}{\sqrt{2\sinh\left[\frac{n\sigma_0^2}{2\sigma_E^2}\right]}}+\left(\frac{\sigma_0\sqrt{c(S-1)}e^{\frac{n\sigma_0^2}{4\sigma_E^2}}}{\sqrt{2\sinh\left[\frac{n\sigma_0^2}{2\sigma_E^2}\right]}}\right)^3\left(\frac{(n-1)^3e^{-\frac{n\sigma_0^2}{\sigma_E^2}}}{2d^2n^2}\right)<m \tag{4.15}$$

Inequality (4.15) is less conservative (i.e. more accurate) than inequality (4.9) because the following inequality always holds for positive x, y and z (it proves that sometimes inequality [4.15] can be true while inequality [4.9] is false):

$$\frac{xe^{\frac{x^2}{4y^2}}}{\sqrt{2\sinh\left[\frac{x^2}{2y^2}\right]}}+\left(\frac{xe^{\frac{x^2}{4y^2}}}{\sqrt{2\sinh\left[\frac{x^2}{2y^2}\right]}}\right)^3ze^{-\frac{x^2}{y^2}}<y+y^3z+x \tag{4.16}$$

For small σ_E, inequality (4.15) yields inequality (2.6):

$$\sigma_0\sqrt{c(S-1)}<m \tag{4.17}$$

For small σ_0, inequality (4.15) yields inequality (1.7):

$$\sigma_E\sqrt{\frac{c(S-1)}{n}}+\left(\sigma_E\sqrt{\frac{c(S-1)}{n}}\right)^3\left(\frac{(n-1)^3}{2d^2n^2}\right)<m$$

4.2.3 Variance-covariance interpretation of the criteria

Current formulation of $\mathbf{A}$ yields a total variance in patch k equal to:

$$\mathbb{V}\left[\mathbf{A}_k\right] = \mathbb{V}\left[a_{ijk}\right] = \mathbb{E}\left[a_{ijk}^2\right] = c\left(1 - \frac{1}{S}\right)\left(\sigma_0^2 + \sigma_E^2\right) \tag{4.18}$$

and a covariance across sites equal to:

$$\mathbb{C}\left[\mathbf{A}_k, \mathbf{A}_l\right] = \mathbb{C}\left[a_{ijk}, a_{ijl}\right] = \mathbb{E}\left[a_{ijk}a_{ijl}\right] = c\left(1 - \frac{1}{S}\right)\sigma_0^2 \tag{4.19}$$

The variance of the average over $\mathbf{A}$, $\overline{\mathbf{A}} = \frac{1}{n}\sum_k \mathbf{A}_k$ is given by:

$$\mathbb{V}\left[\overline{\mathbf{A}}\right] = \frac{1}{n^2}\mathbb{V}\left[\sum_k \mathbf{A}_k\right] = \frac{1}{n}\mathbb{V}\left[\mathbf{A}_k\right] + \frac{n-1}{n}\mathbb{C}\left[\mathbf{A}_k, \mathbf{A}_l\right] \tag{4.20}$$

Plugging equations (4.18) and (4.19) into equation (4.20) yields:

$$\mathbb{V}\left[\overline{\mathbf{A}}\right] = c\left(1 - \frac{1}{S}\right)\left(\sigma_0^2 + \frac{\sigma_E^2}{n}\right) \tag{4.21}$$

Thus, the conservative stability criterion given in inequality (4.9), taken at order 0 in $1/d$ for large d, can be rewritten as :

$$\sqrt{S\mathbb{V}\left[\overline{\mathbf{A}}\right]} < m \tag{4.22}$$

or equivalently, using the correlation coefficient $\rho = \mathbb{C}\left[\mathbf{A}_k, \mathbf{A}_l\right]/\sqrt{\mathbb{V}\left[\mathbf{A}_k\right]\mathbb{V}\left[\mathbf{A}_l\right]} = \sigma_0^2/\left(\sigma_0^2 + \sigma_E^2\right)$:

$$\sqrt{S\mathbb{V}\left[\mathbf{A}_k\right]\left(\frac{1}{n} + \frac{n-1}{n}\rho\right)} < m \tag{4.23}$$

At second order in $1/d$, inequality (4.9) can be rewritten as:

$$\sqrt{S\mathbb{V}\left[\mathbf{A}_k\right]\left(\frac{1}{n} + \frac{n-1}{n}\rho\right)} + \left(S\left(1-\rho\right)\mathbb{V}\left[\mathbf{A}_k\right]\right)\sqrt{S\mathbb{V}\left[\mathbf{A}_k\right]\left(\frac{1}{n} - \frac{\rho}{n}\right)}\left(\frac{(n-1)^3}{2d^2n^3}\right) < m \tag{4.24}$$

A similar reformulation of inequality (4.15) yields the asymptotically exact stability criterion at order 2:

$$\sqrt{\frac{S\rho\mathbb{V}\left[\mathbf{A}_k\right]e^{\frac{n\rho}{2(1-\rho)}}}{2\sinh\left[\frac{n\rho}{2(1-\rho)}\right]}} + \left(\sqrt{\frac{S\rho\mathbb{V}\left[\mathbf{A}_k\right]e^{\frac{n\rho}{2(1-\rho)}}}{2\sinh\left[\frac{n\rho}{2(1-\rho)}\right]}}\right)^3\left(\frac{(n-1)^3e^{-\frac{n\rho}{(1-\rho)}}}{2d^2n^2}\right) < m \tag{4.25}$$

4.3 Stability criterion at small d

To obtain a more precise picture of what happens at small d for any value of ρ, a possibility is to take calculations back from equation (4.14) and to develop the admissibility criterion for small d. Such calculations yield the following admissibility criterion:

$$\frac{(m+z)^2}{(m+z)^2 - \sigma_0^2c(S-1)}\left[1 - \frac{2\sigma_0^2c(S-1)}{(m+z)\left[(m+z)^2 - \sigma_0^2c(S-1)\right]}d\right] > e^{\sigma_0^2/\sigma_E^2} \tag{4.26}$$

Substituting 0 for z and looking at the conditions under which zero is not admissible, we find:

$$\sqrt{\frac{\sigma_0^2c(S-1)e^{\sigma_0^2/\sigma_E^2}}{e^{\sigma_0^2/\sigma_E^2} - 1}}\left(1 - \frac{\sigma_0^2c(S-1)}{m\left[m^2 - \sigma_0^2c(S-1)\right]\left(e^{\sigma_0^2/\sigma_E^2} - 1\right)}d\right) < m \tag{4.27}$$

Now, substituting $\rho/(1-\rho) = \sigma_0^2/\sigma_E^2$ and $\sigma^2 = \sigma_0^2 + \sigma_E^2$, we obtain the equivalent stability condition:

$$\sigma\sqrt{c(S-1)} < \sqrt{\frac{e^{\rho/(1-\rho)} - 1}{\rho e^{\rho/(1-\rho)}}}\left(m + \frac{\rho\sigma^2c(S-1)d}{\left(e^{\rho/(1-\rho)} - 1\right)\left[m^2 - \rho\sigma^2c(S-1)\right]}\right) \tag{4.28}$$

This equation can be further simplified by assuming that the criterion can be written as $\sigma\sqrt{c(S-1)} < \epsilon m + \gamma d$ at low d and solve for the lowest values of ϵ and γ. The equation obeyed by ϵ is:

$$\left(1 - \frac{\epsilon}{\alpha}\right)\left(1 - \rho\epsilon^2\right) = 0 \tag{4.29}$$

where $\alpha = \sqrt{\frac{e^{\rho/(1-\rho)}-1}{\rho e^{\rho/(1-\rho)}}}$. Thus, either $\epsilon = 1/\sqrt{\rho}$ or $\epsilon = \alpha$. However, it is simple to show that $\alpha < 1/\sqrt{\rho}$ in all cases, so we settle for $\epsilon = \alpha$.

The equation obeyed by γ is given by:

$$\left[\frac{3\epsilon^2\rho}{\alpha} - 2\rho\epsilon - \frac{1}{\alpha}\right]\gamma + \beta\epsilon^2 = 0 \tag{4.30}$$

where $\beta = \rho/\left(e^{\rho/(1-\rho)} - 1\right)$. Plugging $\epsilon = \alpha$ and solving for γ, we find $\gamma = \alpha^3\beta/(1-\alpha^2\rho)$. Thus, the final stability criterion at low d becomes:

$$\sigma\sqrt{c(S-1)} < \sqrt{\frac{e^{\rho/(1-\rho)}-1}{\rho e^{\rho/(1-\rho)}}}\,(m+d) \tag{4.31}$$

The coefficient in front of $m + d$ varies between 1 and 1.14 when ρ stays between 0 and 1, thus leading to an approximate criterion in the form of equation (1.8). This coefficient equals 1 for $\rho = 0$ and for $\rho = 1$, and is maximal for $\rho \approx 0.65$.

5 A closer look at the eigenvalues of D

By virtue of the eigenvalue pushback effect, increasing dispersal effectively makes the system more stable. However, we have only looked at a dispersal matrix with global species-independent dispersal. A more general formulation for a symmetric matrix $\mathbf{D}$ yields the following non-zero entries when species i has diffusion rate d_i and diffuses reciprocally with $2v_i$ patches (the 2 is necessary to obtain fully reciprocal diffusion):

$$\begin{aligned} \forall i \in [1;S], \forall k \in [1;n], d_{iikk} &= -2v_i d_i \\ \forall i \in [1;S], \forall k \in [1;n], \forall l (\neq k) \in [k - v_i; k + v_i] \,(\mathrm{mod}\, n), d_{iikl} &= d_i \end{aligned} \tag{5.1}$$

Another way to visualize this definition of $\mathbf{D}$ is to introduce the $S \times S$ diagonal sub-matrices $\mathbf{X}_k$ and $\mathbf{Y}$ defined as:

$$\mathbf{X}_k = \begin{pmatrix} (1 - \delta_{v_1<k<n-v_1})\, d_1 & 0 & 0 \\ 0 & ... & 0 \\ 0 & 0 & (1 - \delta_{v_S<k<n-v_S})\, d_S \end{pmatrix} \tag{5.2}$$

$$\mathbf{Y} = \begin{pmatrix} 2v_1 d_1 & 0 & 0 \\ 0 & ... & 0 \\ 0 & 0 & 2v_S d_S \end{pmatrix} = \sum_{k=1}^{n-1} \mathbf{X}_k \tag{5.3}$$

where δ is Kronecker symbol (equal to 1 when the underlying condition is true, 0 otherwise). Then $\mathbf{D}$ is given by:

$$\mathbf{D} = \begin{pmatrix} -\mathbf{Y} & \mathbf{X}_{n-1} & ... & \mathbf{X}_1 \\ \mathbf{X}_1 & -\mathbf{Y} & \mathbf{X}_{n-1} & \ddots \\ \vdots & \mathbf{X}_1 & \ddots & \ddots \\ \mathbf{X}_{n-1} & \ddots & \ddots & -\mathbf{Y} \end{pmatrix} \tag{5.4}$$

By re-arranging indices in matrix $\mathbf{D}$ so that the first n rows and columns describe the dispersal of species 1, the rows and columns from $n+1$ to $2n$ describe the dispersal of the second species, etc., the following congruence relationship emerges:

$$\mathbf{D} \equiv \begin{pmatrix} \mathbf{D}_1 & \mathbf{0} & \mathbf{0} \\ \mathbf{0} & \ddots & \mathbf{0} \\ \mathbf{0} & \mathbf{0} & \mathbf{D}_S \end{pmatrix} \tag{5.5}$$

where the $n \times n$ sub-matrix block $\mathbf{D}_k$ is described by:

$$\mathbf{D}_k = \begin{pmatrix} -2v_k d_k & (1-\delta_{v_k<n-1<n-v_k})\, d_k & \cdots & (1-\delta_{v_k<1<n-v_k})\, d_k \\ (1-\delta_{v_k<1<n-v_k})\, d_k & -2v_k d_k & \ddots & \vdots \\ \vdots & \ddots & \ddots & (1-\delta_{v_k<n-1<n-v_k})\, d_k \\ (1-\delta_{v_k<n-1<n-v_k})\, d_k & \cdots & (1-\delta_{v_k<1<n-v_k})\, d_k & -2v_k d_k \end{pmatrix} \tag{5.6}$$

$\mathbf{D}_k$ is a symmetric circulant matrix (i.e. arranged by n-periodic bands) with $2v_k + 1$ non-zero entries per row and per column. Using the same congruence transform, we have:

$$\mathbf{D} - \mathbf{M} \equiv \begin{pmatrix} \mathbf{D}_1 - m\mathbf{I}_n & \mathbf{0} & \mathbf{0} \\ \mathbf{0} & \ddots & \mathbf{0} \\ \mathbf{0} & \mathbf{0} & \mathbf{D}_S - m\mathbf{I}_n \end{pmatrix} \tag{5.7}$$

Recalling the general form of inequality (1.6), we know that the limit support of $\mathbf{J}$ depends on the ESD of its deterministic part (i.e. $\mathbf{D} - \mathbf{M}$), so that if $\{\lambda_1, \lambda_2, ..., \lambda_L\}$ are the eigenvalues of $\mathbf{D} - \mathbf{M}$ with multiplicities $\{\omega_1, \omega_2, ..., \omega_L\}$ (with $\sum_k \omega_k = nS$) and $\mathbf{A}$ is heterogeneous among patches (with variance $c\sigma^2$), then the limit support of the ESD of $\mathbf{J}/\sigma\sqrt{cS}$ is given by:

$$\int |z-u|^{-2}\, d\mu(u) = \frac{1}{nS} \sum_{k=1}^{L} \frac{\omega_k}{|z-\lambda_k|^2} \geq 1 \tag{5.8}$$

Because all the eigenvalues of a Hermitian matrix are real, the stability criterion becomes:

$$\mathrm{Sup}\left[z \in \mathbb{R} \,\middle|\, \frac{\sigma^2 c}{n} \sum_{k=1}^{L} \frac{\omega_k}{(z-\lambda_k)^2} \geq 1 \right] < 0 \tag{5.9}$$

In the case of matrix $\mathbf{D}_k - m\mathbf{I}_n$, its eigenvalues, noted $(\zeta_{jk})_{j\in[0;n-1]}$, are real and given by:

$$\begin{aligned} \zeta_{jk} &= -2v_k d_k - m + \sum_{l=1}^{n-1} (1-\delta_{v_k<l<n-v_k})\, d_k e^{-\frac{2i\pi jl}{n}} \\ &= -m - 4d_k \sum_{l=1}^{v_k} \left[\sin\left(\frac{\pi jl}{n}\right)\right]^2 \end{aligned} \tag{5.10}$$

For all k, $\zeta_{0k} = -m$. Based on Gershgorin circle theorem, $-m$ is also the highest possible eigenvalue of $\mathbf{D}_k - m\mathbf{I}_n$, and its multiplicity is necessarily 1 (to have $\zeta_{jk} = -m$, you need $\sin\left(\frac{2\pi jl}{n}\right) = 0$ for all $l \in [1; v_k]$, meaning that $jl \equiv 0 \bmod(n)$ for all $l \in [1; v_k]$, and thus $j = 0$ is the only admissible solution).

Merging the spectra of all $\mathbf{D}_k - m\mathbf{I}_n$, inequality (5.9) becomes:

$$\mathrm{Sup}\left[z \in \mathbb{R} \,\middle|\, \frac{\sigma^2 c}{n} \sum_{k=1}^{S} \sum_{j=1}^{n-1} \frac{1}{\left(z + m + 4d_k \sum_{l=1}^{v_k} \left[\sin\left(\frac{\pi jl}{n}\right)\right]^2\right)^2} + \frac{\sigma^2 cS}{n\,(z+m)^2} \geq 1 \right] < 0 \tag{5.11}$$

One quick and dirty way to get a conservative upper bound for the admissible z in equation (5.11) is to lump the mass of the ESD of each $\mathbf{D}_k - m\mathbf{I}_n$ at $-m$ (with mass $1/n$) and at the second highest eigenvalue (with mass $(n-1)/n$). To do that, we need a lower bound for $\sum_{l=1}^{v_k} \left[\sin\left(\frac{\pi jl}{n}\right)\right]^2$ valid for all $j \neq 0$. Let $\gamma_k > 0$ be such a lower bound and let $d_0\gamma_0 = \underset{k}{\mathrm{Min}}\,[d_k\gamma_k]$. The implied stability criterion becomes:

$$\mathrm{Sup}\left[z \in \mathbb{R} \,\middle|\, \frac{\sigma^2 cS(n-1)}{n\,(z+m+4d_0\gamma_0)^2} + \frac{\sigma^2 cS}{n\,(z+m)^2} \geq 1 \right] < 0 \tag{5.12}$$

Geometrically, if the point z_0 on the real axis and larger than $-m-4d_0\gamma_0$ where $\sigma^2 cS(n-1)/n\,(z_0+m+4d_0\gamma_0)^2 = 1/2$ is lower than the point z_m on the real axis and lower than $-m$ where $\sigma^2 cS/n\,(z_m+m)^2 = 1/2$, then the two

disks (the one centered on $-m$ and the one centered on $-m - 4d_0\gamma_0$) have null intersection (separability condition) and the stability criterion becomes approximatively

$$\left[\frac{1}{\sqrt{n}} + \frac{n-1}{32(d_0\gamma_0)^2 n^{3/2}}\right] \sigma\sqrt{cS} < m \tag{5.13}$$

for high $d_0\gamma_0$. Solving equations for z_0 and z_m, we find that separability occurs when

$$\frac{\sigma\sqrt{2cS}}{4}\left[\sqrt{1-\frac{1}{n}} + \sqrt{\frac{1}{n}}\right] < d_0\gamma_0 \tag{5.14}$$

Another more subtle approximation may be obtained if we assume that all $d_k\gamma_k$ are large. Taking a first possible order approximation, we obtain:

$$\sum_{k=1}^{S}\sum_{j=1}^{n-1} \frac{1}{\left(z + m + 4d_k \sum_{l=1}^{v_k}\left[\sin\left(\frac{\pi j l}{n}\right)\right]^2\right)^2} \approx \sum_{k=1}^{S}\sum_{j=1}^{n-1} \frac{1}{16 d_k^2 \left[\sum_{l=1}^{v_k}\left[\sin\left(\frac{\pi j l}{n}\right)\right]^2\right]^2} \tag{5.15}$$

Based on equation (5.15), plugged in inequality (5.11), the stability criterion is given by:

$$\left[\frac{1}{\sqrt{n}} + \frac{1}{32 n^{3/2} S}\sum_{k=1}^{S}\sum_{j=1}^{n-1} \frac{1}{d_k^2\left[\sum_{l=1}^{v_k}\left[\sin\left(\frac{\pi j l}{n}\right)\right]^2\right]^2}\right]\sigma\sqrt{cS} < m \tag{5.16}$$

which is consistent with, but slightly more precise than, inequality (5.13).

In case $d_k = d$ and $v_k = v$ for all species, inequality (5.16) boils down to:

$$\left[\frac{1}{\sqrt{n}} + \frac{1}{32 d^2 n^{3/2}}\sum_{j=1}^{n-1} \frac{1}{\left[\sum_{l=1}^{v}\left[\sin\left(\frac{\pi j l}{n}\right)\right]^2\right]^2}\right]\sigma\sqrt{cS} < m \tag{5.17}$$

With some algebra

$$\begin{aligned}
\sum_{j=1}^{n-1} \frac{1}{\left[\sum_{l=1}^{v}\left[\sin\left(\frac{\pi j l}{n}\right)\right]^2\right]^2} &= 16\sum_{j=1}^{n-1} \frac{1}{\left[1 - \frac{\sin\left(\frac{\pi j(2v+1)}{n}\right)}{\sin\left(\frac{\pi j}{n}\right)} + 2v\right]^2} \\
&= \frac{16}{(1+2v)^2}\sum_{j=1}^{n-1} \frac{1}{\left[1 - \frac{\sin\left(\frac{\pi j(2v+1)}{n}\right)}{(1+2v)\sin\left(\frac{\pi j}{n}\right)}\right]^2} \\
&= \frac{16}{(1+2v)^2}\sum_{j=1}^{n-1}\sum_{k=0}^{\infty} \frac{1+k}{(1+2v)^k}\left[\frac{\sin\left(\frac{\pi j(2v+1)}{n}\right)}{\sin\left(\frac{\pi j}{n}\right)}\right]^k
\end{aligned} \tag{5.18}$$

Thus, inequality (5.17) is approximately (low order in $1/(1+2v)$):

$$\left[\frac{1}{\sqrt{n}} + \frac{n-1}{2d^2 n^{3/2}(1+2v)^2}\right]\sigma\sqrt{cS} < m \tag{5.19}$$

which is equivalent to inequality (1.7) when $1 + 2v = n$.

Supplementary References

[1] Allesina, S., Tang, S., 2012. Stability criteria for complex ecosystems. Nature 483, 205–208.

[2] May, R. M., 1972. Will a large complex system be stable? Nature 238, 413–414.

[3] Tao, T., Vu, V., Krishnapur, M., 2010. Random matrices: Universality of esds and the circular law. Annals of Probability 38 (5), 2023–2065.
